# Non-Nucleoside Agonists of the Adenosine Receptors: An Overview

**DOI:** 10.3390/ph12040150

**Published:** 2019-10-08

**Authors:** Diego Dal Ben, Catia Lambertucci, Michela Buccioni, Aleix Martí Navia, Gabriella Marucci, Andrea Spinaci, Rosaria Volpini

**Affiliations:** School of Pharmacy, Medicinal Chemistry Unit, University of Camerino, 62032 Camerino, MC, Italy; diego.dalben@unicam.it (D.D.B.); catia.lambertucci@unicam.it (C.L.); michela.buccioni@unicam.it (M.B.); aleix.martinavia@unicam.it (A.M.N.); gabriella.marucci@unicam.it (G.M.); andrea.spinaci@unicam.it (A.S.)

**Keywords:** purinergic receptors, adenosine receptors, adenosine receptor agonists, non-nucleoside agonists, pyridine derivatives, pyrimidine derivatives, ligand–target interaction, drug discovery

## Abstract

Potent and selective adenosine receptor (AR) agonists are of pharmacological interest for the treatment of a wide range of diseases and conditions. Among these derivatives, nucleoside-based agonists represent the great majority of molecules developed and reported to date. However, the limited availability of compounds selective for a specific AR subtype (i.e., A_2B_AR) and a generally long and complex synthetic route for largely substituted nucleosides are the main drawbacks of this category of molecules. Non-nucleoside agonists represent an alternative set of compounds able to stimulate the AR function and based on simplified structures. This review provides an updated overview on the structural classes of non-nucleoside AR agonists and their biological activities, with emphasis on the main derivatives reported in the literature. A focus is also given to the synthetic routes employed to develop these derivatives and on molecular modeling studies simulating their interaction with ARs.

## 1. Introduction

The endogenous nucleoside adenosine regulates a number of physiological and pathological processes through the stimulation of membrane proteins named adenosine receptors (ARs). These proteins are G protein-coupled receptors cloned as four subtypes (A_1_, A_2A_, A_2B_, and A_3_ ARs) [1,2] and coupled to cytoplasmic GTP-binding proteins that mediate the intracellular effects of AR stimulation. In particular, the stimulation of the A_2A_ and A_2B_ ARs leads to an increase of intracellular cAMP levels through activation of a G protein, while the stimulation of the A_1_ and A_3_ ARs leads to a decrease of intracellular cAMP levels through activation of Gi/o proteins. Modulation of further second messengers has also been described, which involves the activation of signaling mechanisms like phospholipase C, Protein Kinase C (PKC), phosphoinositide 3-kinases, ion channels, and the modulation of calcium levels [1,3,4]. The stimulation of the various AR subtypes has effects on Central Nervous System (CNS) and peripheral tissues [5,6], modulating release of neurotransmitters, synaptic transmission [7], heart rate and atrial contractility, vascular smooth muscle tone [8], gastrointestinal functions [9,10], as well as renal [11], platelet [12], and leukocyte [13] functions. Therefore, AR regulation represents a high-potential strategy for the development of therapeutic tools. On the other hand, widespread AR expression requires compounds endowed not only with high potency and efficacy at the various ARs but also with selectivity for specific AR subtypes [14]. To date, a limited number of adenosinergic ligands have been approved for therapy besides Ado itself, that is, the A_2A_AR agonist Regadenoson (approved as a coronary vasodilator) and the A_2A_AR antagonist Istradefylline as an anti-Parkinson drug [15,16,17].

Several compounds have been designed and developed as AR agonists, where adenosine generally represents the core scaffold further modified to obtain compounds with various degrees of AR potency and selectivity [18]. The key modifications made to the endogenous ligand are at both the purine moiety and the ribose ring. In the first case, substituents introduced at the 2- and *N*^6^-position lead to an improvement of the affinity/potency and often selectivity for specific AR subtypes, depending on the volume and chemical-physical profile of the substituents themselves. A key modification of the ribose moiety is at its 4’-position, with the introduction of an *N*-alkylcarboxamido function (the so-called MECA, N-methylcarboxamidoadenosine, or NECA, N-ethylcarboxamidoadenosine, derivatives, where the alkyl group consists of a methyl or an ethyl function, respectively) [19,20,21,22]. This modification generally improves the activity at all the AR subtypes with respect to the corresponding adenosine analogues. Conversely, the removal of the ribose moiety or its replacement with a small alkyl group was generally observed as associated to an agonist-to-antagonist switch of the pharmacological profile [23,24,25].

Recent developments in this field have occurred since the publication of some patents and articles in the early 2000s describing non-nucleoside agonists of the ARs [26,27,28,29,30]. The early data are related to non-selective agonists ranging from partial to full agonist profiles. Subsequent reports described further non-nucleoside derivatives endowed with low nanomolar potency and improved selectivity for the A_1_, A_2A_, or A_2B_ AR subtypes [30,31,32,33,34,35,36,37,38]. Structural features and biological activity of these molecules, synthetic approaches, and molecular modelling studies simulating the interaction between these compounds and AR targets are reviewed in this work.

## 2. Non-Nucleoside Agonists of the ARs

### 2.1. Structural Features and Biological Activity

#### 2.1.1. Pyridine Derivatives

**Figure 1:**
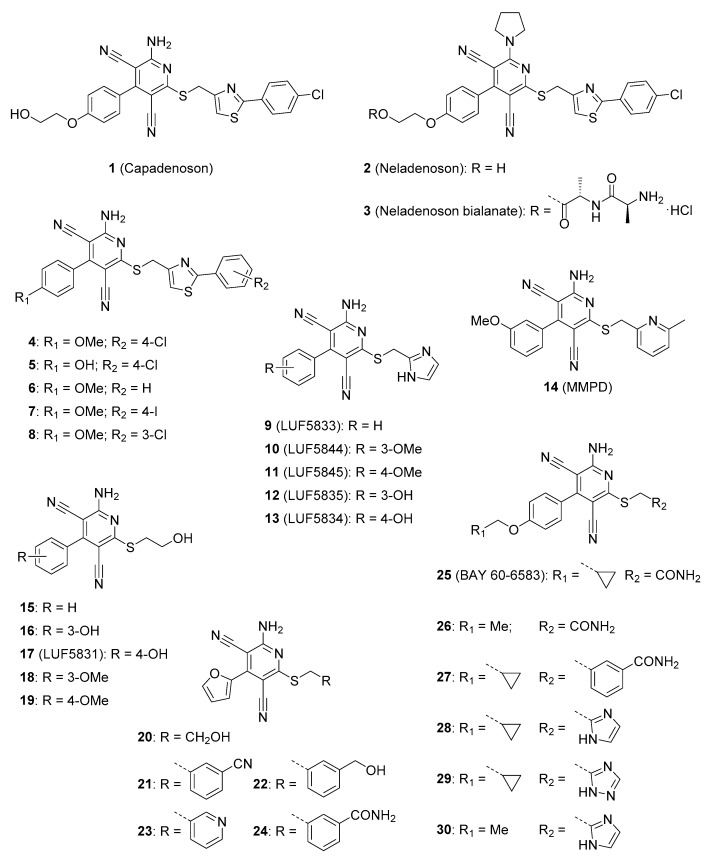
Selected pyridine-based non-nucleoside agonists of the adenosine receptors.

The discovery and development of pyridine-based non-nucleoside agonists of the ARs started from the publication of patents from Bayer describing pyridine derivatives endowed with agonist activity at the ARs [26,27,28,29,39]. These compounds were generally 2-aminopyridines presenting two cyano groups at the 3- and 5-positions, a phenyl group in the 4-position, and in the 6-position a further substituent starting with a methylthio spacer followed by groups of various volumes and chemical-physical properties. The key structural modifications applied to the series were related to the 6-chain and to further substituents to be inserted to the 4-phenyl ring. These studies led to the discovery of compounds of significative importance, in particular for the A_1_AR. One of these compounds is the A_1_AR agonist 2-amino-6-[[2-(4-chlorophenyl)-1,3-thiazol-4-yl]methylsulfanyl]-4-[4-(2-hydroxyethoxy)phenyl]pyridine-3,5-dicarbonitrile, also named BAY 68-4986 or Capadenoson (**1**; Figure 1; Table 1). This compound consists in a 2-aminopyridine presenting two cyano groups at the 3- and 5-positions, a substituted phenyl group in the 4-position, and in the 6-position a thiomethyl spacer with a substituted thiazole. Capadenoson currently represents a reference A_1_AR partial agonist, endowed with subnanomolar potency at the human A_1_AR [39,40,41]. Capadenoson was shown to dose-dependently modulate the stress-induced heart rate changes and the release of norepinephrine from cardiac presynaptic nerves in the perfused hearts of spontaneously hypertensive rats [42]. In canine models of heart failure, the administration of Capadenoson improved the left ventricular function and prevented progressive remodeling [43]. In a phase II clinical study, Capadenoson was shown to modulate the heart rate in patients with stable angina [44], even if it was later withdrawn [45]. In another phase II clinical study (patients with persistent or permanent atrial fibrillation), this molecule did not show a relevant effect in the modulation of heart rate [46,47]. Further clinical evaluations of this molecule were not reported. Capadenoson was also studied as modulator of body temperature in rats, showing a slight hypothermic effect compared to the A_1_AR full agonist CHA [48]. This molecule also showed a favorable DMPK (drug metabolism and pharmacokinetics) profile [39,46] The advantages of the use of partial A_1_AR agonists were highlighted in one of these works, including the ability of these compounds to not lead to receptor desensitization, the multifaceted set of effects in various tissues where different levels of A_1_AR expression are observed, the lower risk of producing severe side effects with respect to full agonists [46]. A biased agonism of Capadenoson was also assessed since this compound exhibited high potency in the activation of all the intracellular pathways upon A_1_AR stimulation, with the exception of the intracellular calcium mobilization, where the effect was lower with respect to other A_1_AR reference agonists [49]. Considering the AR affinity, Capadenoson showed also a high A_1_AR selectivity versus the other AR subtypes where the percentage of radioligand displacement was 0–2.5% [36]. In contrast, a recent work reported that this molecule can also bind the A_2B_AR with a Ki of about 300 nM, but the authors did not specify the affinity at the other subtypes. In the same work, the potency of **1** in functional experiments was given as EC_50_ equal to 0.66 nM, 1400 nM, and 1.1 nM at the A_1_, A_2A_, and A_2B_ ARs, respectively (Table 1) [41].

Interestingly, Capadenoson showed a biased agonism also at the A_2B_AR since it activated the cAMP pathway with higher potency with respect to the other pathways induced by A_2B_AR stimulation. Structural modifications at the exocyclic amine group of Capadenoson led to the development of the A_1_AR agonist 2-(((2-(4-chlorophenyl)-1,3-thiazol-4-ylmethyl)sulfanyl)-4-(4-(2-hydroxyethoxy)phenyl)-6-(pyrrolidin-l-yl)pyridine-3,5-dicarbonitrile, also named Neladenoson (**2**; Figure 1; Table 1). Like Capadenoson, Neladenoson is a selective partial agonists of the A_1_AR [50,51]. Neladenoson showed cardioprotective effects in rat preclinical models, analogously to Capadenoson, although with lower central effects. A bialanate (alanine–alanine ester) derivative of Neladenoson (in its hydrochloride salt form) was also developed as a prodrug, showing a more favorable pharmacokinetic profile. 

Neladenoson bialanate (BAY 1067197; **3**; Figure 1) is currently in clinical evaluation for the treatment of chronic heart failure with preserved/reduced ejection fraction (HFpEF and HFrEF, respectively) [50,51,52,53,54,55,56,57]. Early results show a lack of dose-dependent favorable effects on cardiac structure and function and on exercise capacity, with a dose-dependent decrease in renal function [58,59].

Further derivatives of Capadenoson were developed, with the evaluation of their binding affinity and dissociation kinetics properties at the A_1_AR (as examples are reported compounds **4–8**; Figure 1; Table 1). Several of these derivatives were endowed with good A_1_AR affinity and selectivity, ranging from partial to full agonist profiles [36]. In particular, considering the modifications on the 4-phenyl ring of Capadenoson, the replacement of the para-hydroxyethyloxy substituent with a hydroxy group maintain the A_1_AR affinity of Capadenoson (**5**, Ki A_1_AR = 1.5 nM, versus **1**, Ki A_1_AR = 1.4 nM; Table 1). The replacement of the same moiety with a methoxy group (**4**) led to the synthesis of a Capadenoson analogue that was further modified at the phenyl group present in the 2-position of the thiazole ring. In this set of derivatives, high A_1_AR affinities were obtained with compounds presenting a phenyl ring substituted in the para-position with a halogen atom (i.e., **7**). In some cases, compounds were found endowed with residence times up to 132 min (**4**), about 4.7-fold longer than Capadenoson itself [36]. 

A work by Beukers and colleagues [31] described five 2-aminopyridines derivatives with similarities to Capadenoson, given the presence of two cyano groups at the 3- and 5-positions and a phenyl group in the 4-position. In the 6-position, a thiomethyl spacer with a 2-imidazole ring was inserted. The 4-phenyl group could be further substituted with a hydroxy or methoxy group in the para- or meta-position. Results of biological evaluation of these compounds showed a low nanomolar affinity for the A_1_ but also for the A_2B_ ARs (**9–13**; Figure 1; Table 1). The most potent A_1_ and A_2B_ AR agonists of this group (then named LUF5844, **10**, and LUF5845, **11**, respectively) present a methoxy group in the meta- or para-position 4-phenyl ring, respectively. A modification of LUF5844 by substituting the imidazole ring with a 6-methylpyridin-2-yl group led to the development of the compound 2-amino-4-(3-methoxyphenyl)-6-(2-(6-methylpyridin-2-yl)ethyl)pyridine-3,5-dicarbo- nitrile (MMPD; **14**; Figure 1; Table 1), a partial A_1_AR agonist endowed with subnanomolar affinity for this receptor and high selectivity versus the other AR subtypes. This compound was also developed as a radioligand useful for Positron Emission Tomography (PET) imaging in the brain, given its good pharmacokinetics profile related to the ability to cross the Blood Brain Barrier (BBB) [60,61]. Even the compound 2-amino-4-(4-hydroxy phenyl)-6-(1H-imidazol-2ylmethylsulfanyl)-pyridine-3,5-dicarbonitrile [31], then named LUF5834 (**13**; Figure 1; Table 1), was developed as a radioligand able to bind to A_1_AR in both G-protein-coupled and uncoupled conditions with a similar high affinity [34]. Since this molecule showed nanomolar affinity also for the A_2A_AR, it was employed for a mutagenesis study at this AR subtype, in comparison with the nucleoside derivative CGS21680. Results showed that the potencies of LUF5834 and CGS21680 are differently subjected to variations due to mutations of binding site residues, suggesting different ligand–receptor interactions between nucleoside and non-nucleoside agonists and the A_2A_AR [37]. The same research group later reported the synthesis and biological evaluation of **9–13** analogues where the imidazolyl-methyl-thio substituent in the 6-position was replaced by a hydroxy-ethyl-thio group [32]. In general, these compounds, (**15–19**; Figure 1; Table 1), showed A_1_AR selectivity, with affinity in the nanomolar range. Low nanomolar or subnanomolar potencies of some of these compounds at the A_1_AR were also reported by Bayer researchers [39]. Interestingly, some of these derivatives showed full agonist activity at the A_1_AR, while other similar derivatives proved to be inverse agonists of the same receptor. The presence of a small substituent in the meta-position of the 4-phenyl ring generally appeared more important with respect to the insertion of the same group in the para-position for the A_1_AR affinity and for both agonist and antagonist/inverse agonist activities [32]. One of these compounds, named LUF5831 (**17**; Figure 1; Table 1), initially showed almost full agonist activity at the A_1_AR. In a subsequent work [33], the A_1_AR partial agonist profile of the same compound was reported. Interestingly, the same molecule appeared to maintain the affinity for a mutated A_1_AR (T277A) differently from the nucleoside agonist cyclopentyladenosine (CPA) that lost its binding ability to the mutated receptor. Furthermore, the affinity of LUF5831 for the A_1_AR was reduced by the presence of the allosteric modulator PD81,723, which conversely led to an improvement of the A_1_AR affinity of the nucleoside agonist CPA [33]. 

This suggested that the interaction between the non-nucleoside agonist LUF5831 and the receptor could involve some residues different with respect to the ones interacting with the nucleoside derivative CPA, as later suggested also at the A_2A_AR (see above LUF5834) [37]. 

Further 2-amino-3,5-dicyanopyridines were recently reported, bearing a 2-furyl ring in the 4-position and a thiomethyl chain in the 6-position, further substituted with groups of various volumes, polarity, and chemical-physical properties. Interestingly, all these compounds showed antagonist activity at the A_1_AR, with affinity data in the low nanomolar range in several cases (as examples are reported compounds **20–24**; Figure 1; Table 1). The replacement of the 4-phenyl ring with a 2-furyl group seems critical for the agonist-to-antagonist shift in the intrinsic activity of the compounds [62].

Among the pyridine derivatives acting as agonists at the ARs, a key compound is BAY 60–6583 (**25**; Figure 1; Table 1). This molecule was introduced as a selective A_2B_AR agonist, with EC_50_ potency data at a low nanomolar level [39,65,66]. Various affinity/potency data were then reported for this molecule at the same receptor, in all cases ranging in the nanomolar/submicromolar level [39,63,64,67,68]. These observed variations of affinity or potency may be due to different assay protocols or conditions. Considering the molecular structure, the 3,5-dicyano-6-aminopyridine nucleus is still present, with the introduction of a substituted phenyl ring in the 4-position and a thioacetamide chain in the 2-position. This molecule soon became a reference ligand for pharmacological studies involving the A_2B_AR, given the low availability of selective ligands for this receptor. In a mutagenesis study at the A_2B_AR, the potency and efficacy of BAY 60–6583 was tested at various receptor mutants, in comparison to the nucleoside agonist NECA [68]. Results showed that in some cases, the mutations led to different effects for non-nucleoside and nucleoside agonists, considering both the potency (EC_50_ data) and efficacy as full or partial agonist, in agreement to what was observed at the A_1_AR [33] and A_2A_AR [37]. The partial agonist profile of this molecule at the A_2B_AR was then assessed [69], even if the results of the study suggested the authors to highlight that the intrinsic activity of this molecule could vary from full agonist to antagonist depending on the tissue, the receptor expression level, and the local adenosine concentration. BAY 60–6583 was used for several pharmacological studies, analyzing in particular its cardioprotective effects [70,71]. Beneficial effects of this molecule for obesity [72], lung injury [73], and insulin resistance [74,75] were also reported. Though this molecule never entered in clinical trials, analogues of BAY 60–6583 were synthesized and tested [64]. These derivatives differed from BAY 60–6583 in terms of the substituent on the 4-phenyl ring or the 2-chain (as examples are reported compounds **26–30**; Figure 1; Table 1). Interestingly, one of these compounds (**28**), bearing a cyclopropylmethyloxy group in the para-position of the 4-phenyl ring (like BAY 60–6583) and a 2-imidazolyl-methyl-thio chain in the 2-position, showed low nanomolar potency in functional assay at the A_2B_AR together with high nanomolar affinity in binding experiments and seems to show an interesting selectivity versus the other AR subtypes. This selectivity is higher compared to analogues bearing the same 2-substituent and presenting in the 4-position a phenyl ring unsubstituted or substituted by smaller groups (**9–13**; Figure 1; Table 1) [31]. Further derivatives of this work showed A_2B_AR potency within the nanomolar range, although in some cases with limited selectivity.

#### 2.1.2. Pyrimidine Derivatives

The pyrimidine scaffold was used as a core structure for the development of AR ligands generally endowed with antagonist activity and, in some cases, low nanomolar affinity [76]. These compounds were 2,6-diphenylpyrimidine or 4,6-diphenylpyrimidine derivatives bearing a substituted amide function in the 4- or 2-position, respectively. The second group showed a marked selectivity for the A_1_AR. Further development of these compounds, obtained by mixing their structural features with the ones of the already described cyanopyridine agonists (see above [31,32]), led to the synthesis of pyrimidine derivatives endowed with inverse agonist activity at the same receptor [77]. The combination of two aromatic rings in the 4- and 5-position of pyrimidine led to the development of an A_2B_AR antagonist [78]. A few years later, with respect to the publication of the early patents on pyridine derivatives, two patents about cyanopyrimidine derivatives as A_2A_AR agonists were reported by Otsuka Pharmaceuticals [30,38]. 

**Figure 2:**
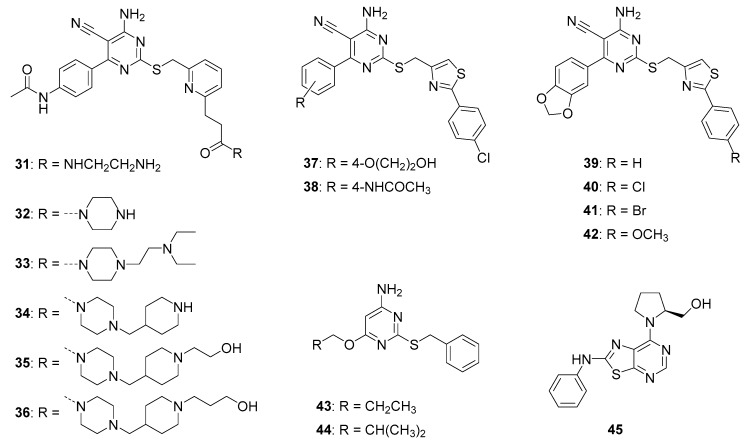
Selected pyrimidine-based non-nucleoside agonists of the adenosine receptors.

These molecules were 4-amino-6-aryl-5-cyano-2-thiopyrimidines, presenting an acetylamino group in the para-position of the 6-phenyl ring and various substituents within the 2-chain (as examples are reported compounds **31–36**; Figure 2; Table 2). These compounds present several similarities compared to the above-described cyanopyridine agonists, with the main difference consisting in the absence of one cyano group of the pyridines substituted by a nitrogen atom in the aromatic scaffold. Several of these compounds were claimed as being endowed with low nanomolar potency at the A_2A_AR. 

In a subsequent work, Louvel and coworkers [35] described cyanopyrimidine obtained by combining structural features of Capadenoson with the pyrimide core. These molecules showed A_1_AR affinity at a low nanomolar level and a good selectivity for the same receptor, with generally a partial agonist profile (as examples are reported compounds **37–42**; Figure 2; Table 2). High A_1_AR affinities were obtained in particular with compounds bearing a 3,4-methylenedioxy substituent on the 4-phenyl ring and a substituted phenyl ring linked to the thiazole moiety of the 2-substituent, with some analogies with respect to what was observed in a study on pyridine-based Capadenoson analogues [36]. In addition, dissociation kinetics were analyzed and reported for the same compounds.

Pyrimidine analogues bearing a 2-thioarylalkyl chain and an alkyloxy group in the 6-position were developed as AR ligands. These compounds (lacking the 5-cyano group and an aromatic ring within the 6-substituent) showed antagonist activity at the ARs [79]. In a successive work, the same authors tested analogues of these molecules based on a pyrimidine scaffold, with the presence of a phenylmethyloxy chain in the 6-position [80]. Biological evaluation of these compounds showed that they are endowed with micromolar affinity for the A_1_AR and high nanomolar agonist potency at the same AR subtype (**43–44**; Figure 2; Table 2). This suggested the importance of the presence of the 5-cyano group and an aromatic ring directly linked to the heterocyclic core to achieve nanomolar affinity at ARs. The pyrimidine core was also fused with a thiazole moiety to obtain thiazolo[5,4-d]pyrimidine agonists of the A_2A_AR. These compounds mimicked some structural features of adenosine, since the 6-substituent inserted in the bicyclic core of the thiazolopyrimidines appears to provide analogue interaction with the receptor cavity with respect to the adenosine ribose ring, according to molecular modelling studies [81]. Results of the biological evaluation of these compounds showed high nanomolar A_2A_AR affinity and moderate selectivity for some derivatives, with a partial agonist profile (as example **45**; Figure 2; Table 2).

### 2.2. Synthetic Approaches

As reported above, two main scaffolds of compounds with non-nucleosidic structure and agonist properties for ARs have been identified. 

**Scheme 1:**
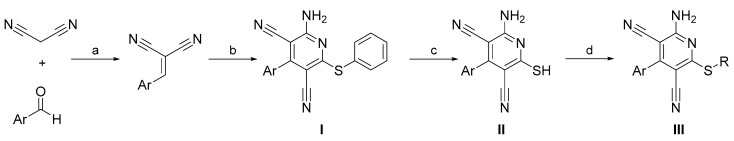
Synthetic route to obtain the 3,5-dicarbonitrile pyridines. Reagents and conditions: (a) piperidine, EtOH; (b) malononitrile, thiophenol, Et_3_N, EtOH; (c) (i) Na_2_S, DMF, (ii) 1 M HCl; (d) alkylhalide, NaHCO_3_, DMF; Ar = aryl, R = alkyl.

The first series of compounds were substituted 3,5-dicarbonitrile pyridine. Their synthesis and biological evaluation were reported in two patents by Bayer [26,27]. The synthetic procedure involved the reaction of an aldehyde with malononitrile in basic conditions to give the aryilidenemalononitrile, which reacted with another molecule of malononitrile and, in the presence of thiophenol, furnished the 2-amino-4-aryl-6-(phenylthio)pyridine-3,5-dicarbonitriles (general structure **I**). The latter compound was used for the production of the useful intermediate 6-thiol derivative **II** by elimination of the phenyl group with sodium sulfide and then treatment with chloridric acid to get the thiol derivative. The reaction of **II** with the suitable alkylhalide chains furnished the desired final 3,5-dicarbonitrile pyridines **III** (Scheme 1). 

**Figure 3:**
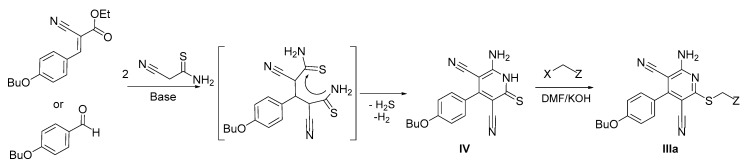
Mechanism proposed by Dychenko and Litvinov for the cyclization formation of 3,5-dicyano pyridine derivatives. X = Br, Cl; Z = 4-Cl-Ph, CH_3_COO, PhCO, CH_2_=CH, NH_2_CO, H, 4-Br-PhCO, 4-Cl-PhCO, EtCOO, PhCH_2_COO, 2-oxo-3-pyrazynylcarbonyl, PhNHCO, CN; B = N-methylmorpholine.

The reaction pathway for the production of such a series of compounds was previously reported by other authors [82,83,84]. In particular Dyachenko and Litvinov obtained the 6-amino-3,5-dicyanopyridine-2(1H)-thione derivatives **IIIa** from ethyl 3-(4-butoxyphenyl)-2-cyanoacrylate or 4-butoxy benzaldehyde and cyanothioacetamide in basic conditions by N-methylmorpholine with the reaction mechanism suggested, as depicted in Figure 3 [85].

Subsequently, on the basis of data previously published in the patent from Bayer, Beukers and coworkers evaluated some 3,5-dicarbonitrile derivatives as human A_2B_ adenosine receptor agonists, which were synthesized with the previously reported procedure [31]. Both the synthetic approaches used the thiol intermediate that was reacted with the suitable organic halide to obtain the final 2-thioalkylpyridine derivatives.

**Scheme 2:**
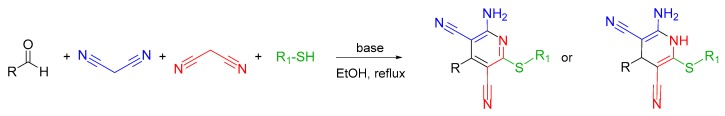
Synthesis of the 2-amino-3,5-dicyano-6-sulfanylpyridines via the single-step, three-component reaction.

A different methodology was used by Evdokimov and colleagues, who prepared a series of 2-amino-3,5-dicyano-6-sulfanylpyridines and 1,4-dihydropyridines via a single-step, three-component reaction of malononitrile with different aldehydes and thiols (Scheme 2) [86]. 

**Figure 4:**
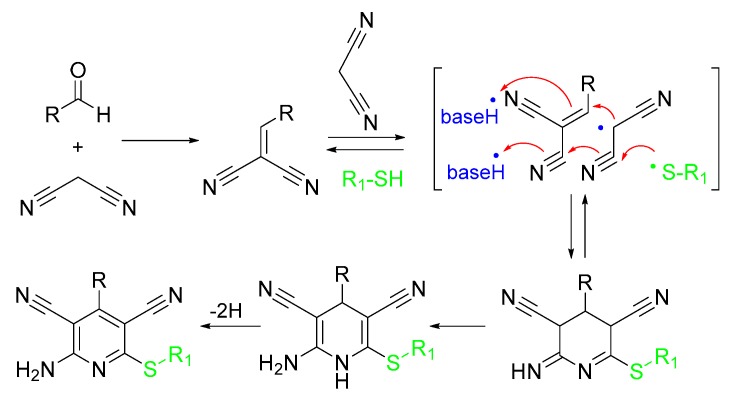
Reaction mechanism of the single-step, three-component reaction formulated by Evdokimov.

The hypothesized mechanism of the reaction includes the formation of 1,4-dihydropyridines, which undergo oxidation by the Knoevenagel adducts rather than air oxygen (Figure 4). As base, 1,4-diazabicyclo[2.2.2]octane (DABCO) or trimethylamine was used and 2-amino-3,5-dicarbonitrile-6-thio-pyridine derivatives in 20–48% yields were obtained due to the formation of 1,4-dihydropyridines as side products. 

Due to the importance such a class of molecules has acquired over the time, several other synthetic routes have been attempted. In particular, in one of them are reported conditions that led to the use of less pollutant reagent/catalyst/waste or better yields using a different catalytic agent.

**Figure 5:**
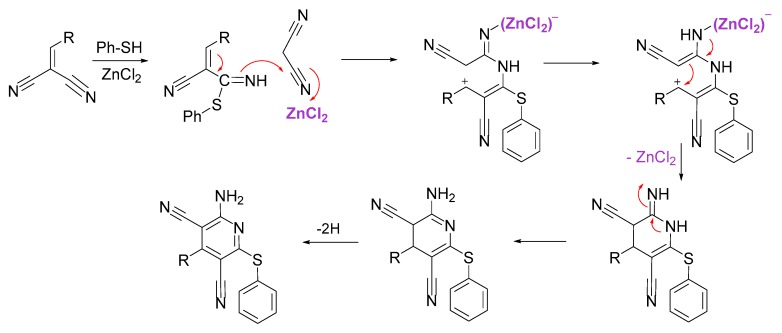
Reaction mechanism theorized when the Lewis acid ZnCl_2_ was used.

Sridhar and co-workers [87] firstly reported the application of Lewis acids as catalysts in the preparation of this class of compounds using the single-step approach to react the suitable aldehyde with malononitrile and thiophenol. Furthermore, a comparison of conventional or microwave heating to obtain the 2-amino-3,5-dicarbonitrile-6-(phenylthio)pyridine-4-substituted intermediates was described. The substitution of the base catalysis with the Lewis acid led to a doubling of the yield of the reaction when ZnCl_2_ was used as Lewis acid (Figure 5). Comparing the two methods, it is possible to assume that there was not an increment of the yield with the microwave protocol, but in this case, a few minutes of heating was sufficient to obtain the final product with good yield.

Subsequently, other authors used the same approach but using different Lewis acids. Kottawar and colleagues used scandium triflate to produce highly substituted pyridines. In this case, the yields of the reaction were subject to the used aldehyde, but they performed the reaction on a great variety of aldehydes to furnish a good library of data in the production of 3,5-dicyanopyridines [88]. The increasingly eco-friendly requirements for synthetic protocols pushed several researchers to also look for new materials and conditions. Takale and co-workers [89] investigated the use of iodoxybenzoic acid (IBX) in aqueous media as an oxidant catalyst. The activity of IBX was compared with other compounds such as β-cyclodextrin, ceric ammonium nitrate or sulfate, tetrabutylammonium hydrogen sulphate, urea hydrogen peroxide, and cupreous chloride at reflux conditions for 3–5 h, but the yields of recovered product were 32–60%, while with IBX at 70 °C, for a 1.5 h reaction time, the yield was 80%. Additionally, the catalyst could be recovered and reused as it maintains its catalytic property. Thimmaiah and colleagues [90] set up a multicomponent reaction for the production of 2-amino-3,5-dicarbonitrile-6-thio-pyridines using heterogeneous catalysis by Zn(II) or a Cd(II) metal–organic framework (MOF). The advantages of the method were the tolerability of different functional groups present on the substrates, the recovery and reuse of the catalysts, and the fact that the reaction does not necessitate the use of any organic solvent. Moreover, in these conditions, the reaction requires less reaction time (30–60 min), with, in general, very high yields of the product isolated by column chromatography. 

Among the nanoparticle materials, CuI nanoparticles were also used as a worthwhile and reusable catalyst supply and eco-friendly procedure for the synthesis of 2-amino-3,5-dicyano-6-sulfanyl pyridine derivatives. The products were obtained, in the better conditions, using CuI with a high specific surface area and approximately crystalline size of 60 nm in ethanol/water under reflux conditions. The reaction took 100 min and via a multicomponent reaction of 4-chlorobenzaaldehyde, malononitrile, and thiophenol under reflux conditions, gave a 90% yield on the isolated product. The method presented is mild, efficient, inexpensive, and satisfactory to give the products in the presence of novel nanoscale materials [91].

Another approach to furnish this class of compounds was the use of ionic liquids. This approach has the advantage of the use of solvents with very low vapor pressure, good thermal stability, and the possibility to recycle and reuse them. The use of the ionic liquids is strategic also because gas, and in particular oxygen, can be present in the solvent at a greater concentration versus other organic solvents, allowing the easy dehydrogenation of 1,4-dihydropyridines leading to the pyridine derivatives by aromatization of the intermediate. Tian and Guo used 1-butyl-3-methylimidazolium tetrafluoroborate ([bmim]BF_4_) as an ionic liquid, which gave a higher yield of 2-amino-4-phenyl-6-(phenylsulfanyl)-3,5-dicyanopyridines with respect to the corresponding chlorine or bromine ionic halide liquids [92]. In this case, the reaction was performed with the three-component approach, aromatic aldehyde, malononitrile, and thiophenol, at 50 °C. The advantages of the method were the very high yields (78–89%), the short reaction time (20–30 min) together with high selectivity and milder reaction conditions, and the recovery and reuse of the solvent. 

**Figure 6:**
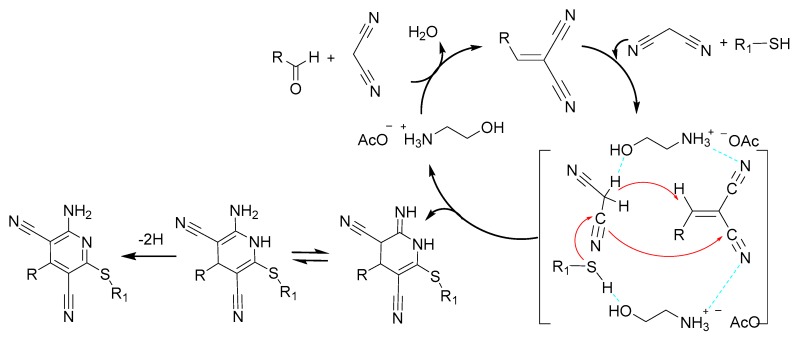
Reaction mechanism hypothesized for the mutual activation of the substrates and the ionic liquid.

Other authors used 2-hydroxyethylammonium acetate (2-HEAA) as an ionic liquid for the multicomponent reaction [93]. By using 2-HEAA, the reaction was kept at room temperature for 5 min. Water was added to work up the reaction mixture from which the product was filtered off and the ionic liquid recovered after evaporation of the filtrate and drying at 100 °C under vacuum. The reuse of the solvents, recovered from a previous reaction batch, led to obtaining the desired product with an almost quantitative yield in the usual time. The authors hypothesized a mutual activation of the substrates and the ionic liquid, as shown in Figure 6.

**Scheme 3:**
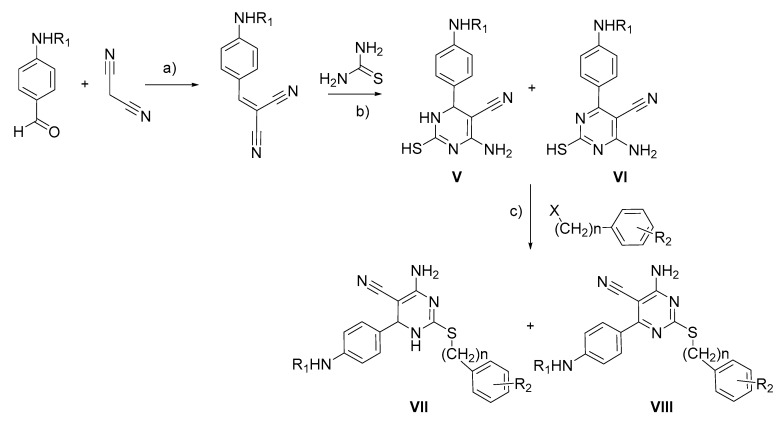
Synthetic route for the pyrimidine derivatives with agonist properties at ARs. Reagents and conditions: a) piperidine, EtOH, r.t.; b) EtONa, EtOH, reflux; c) NaHCO_3_, DMF, r.t.

In 2008, a patent (WO/2005/105778) by Otsuka Pharmaceutical reported the synthesis of 4-amino-5-cyanopyrimidine derivatives as potent A_2A_AR agonists (second series of derivatives). Compounds were obtained through the synthesis of the intermediate 2-mercapto-4-substituted pyrimidine **VI** (Scheme 3). The scaffold was obtained using a previously reported procedure [94], by reaction of the useful substituted benzaldehyde with malononitrile, using ethanol as solvent, and an equimolar amount of an organic base, such as piperidine, at room temperature to give the 2-(4-substitutedbenzylidene)malononitrile. The latter compound was reacted with thiourea [95] in ethanol and in the presence of sodium ethoxide at reflux to obtain a mixture of pyrimidine or dehydropyrimidine derivatives **V** and **VI**. The reaction of the mixture of compounds with the suitable alkyl-aryl-halide in DMF as solvent, and in the presence of sodium bicarbonate as the base at room temperature, furnished the desired final compounds as 2-alkylthiopyrimidines **VIII** or 2-alkylthiodehydropyrimidines **VII**. The dihydro compound **V** can be transformed to the pyrimidine derivative **VI** by an oxidation reaction. 

**Scheme 4:**
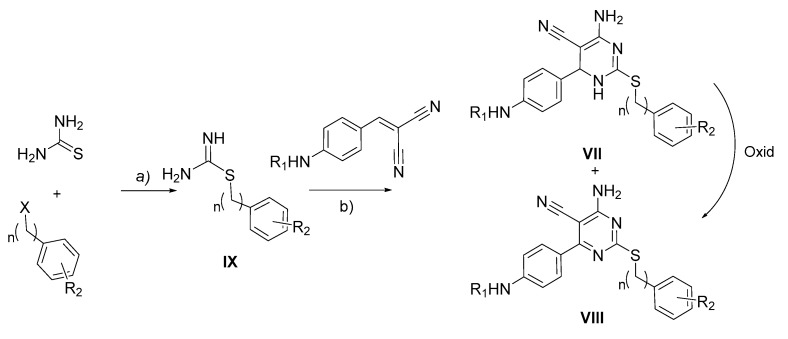
Alternative route for the synthesis of the pyrimidine derivatives. Reagents and conditions: a) base/acid, EtOH, 60 °C; b) NaHCO_3_, EtOH.

Treatment with N-bromosuccinimide (NBS) in ethanol or using 2,3-dichloro-5,6-dicyanobenzoquinone (DDQ) in dioxane at reflux transformed the dehydro derivative **V** in the oxidized compound **VI**. Alternatively, the final compound could also be obtained by reaction of the thiourea with the alkyl-aryl-halide through the S-alkyl isothioureas **IX**. The reaction was performed in ethanol in the presence of a base or an acid at 60 °C. The intermediate was obtained as a free form or a salt form [96]. The S-alkyl isothioureas **IX** was then reacted with the benzylidene-malononitriles in ethanol and in the presence of sodium bicarbonate to furnish the desired compounds **VIII** (Scheme 4) [97].

**Scheme 5:**
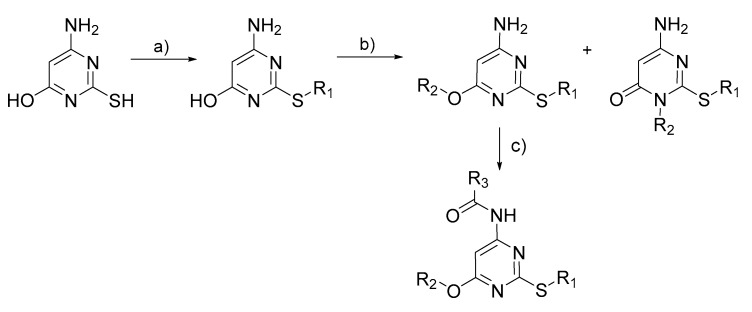
Synthesis for the 6-alkoxy-pyrimidines. Reagents and conditions: a) alkyl/alkyl-aryl halide, 1 M NaOH, 50–55 °C; b) alkyl/alkyl-aryl halide, anhydrous DMF, excess K_2_CO_3_, reflux; c) anhydride, catalytic conc H_2_SO_4_, 50–60 °C.

Starting from 2008, Cosimelli and coworkers published a series of compounds with agonist properties for ARs characterized by a common 6-alkoxypyrimidine scaffold [79,80,98]. The synthesis of such compounds was obtained by alkylation of the sulfur atom in the 2-position of the commercially available 4-amino-6-hydroxy-2-mercaptopyrimidine with the suitable alkyl halide in an aqueous solution of sodium hydroxide (Scheme 5). With the aim at alkylating the oxygen atom at 6-position, the 2-S-alkyl pyrimidines where then reacted with the suitable alkyl halide in DMF and excess of potassium carbonate. The reaction furnished a mixture of O- or N-alkylated derivatives due to the keto-enolic equilibrium forms present of the reagent. Finally, the O-alkylated derivatives were reacted with the opportune anhydride and concentrated sulfuric acids as catalyst.

The identification of non-nucleoside agonists with new scaffolds is very interesting, especially from the synthetic point of view. In fact, before the discovery of the pyridine and pyrimidine derivatives, all AR agonists were adenosine derivatives. Their synthesis is very complex and concerns two different so-called divergent or convergent approaches. In the first, a nucleoside is modified to obtain adenosine derivatives. An example is the synthesis of the full agonist 2-substituted NECA derivatives that can be obtained in about nine steps from guanosine to get the intermediate useful for the synthesis of compounds bearing a substitution in the 2-position, which is important for the selectivity of these compounds versus A_2A_/A_3_ AR subtypes [99]. In the “convergent approach”, the nucleosides are obtained by a glycosylation reaction through a modified nitrogen heterocyclic base and a suitable sugar both obtained after modification of commercially available bases and sugars, in different synthetic steps. The disadvantage of this method is that a mixture of different anomeric and isomeric compounds could be obtained based on coupling conditions, heterocyclic base, and sugar structures and reactivity [100,101]. Basically, nucleoside production consists of several synthetic passages, which involve the purification of the products with relatively complex methods and yields of the final products that could be very low due to the formation of side compounds.

The synthesis of nucleoside analogues furnished very potent and selective AR agonists that are actually used as tools to study ARs, and several examples are in clinical trials but in general with poor pharmacokinetic properties and very complex synthetic routes for their realization [4,14,18,61]. Hence, the need to discover non-nucleosidic scaffolds gives the advantage of low-cost compounds obtained with simple and fast methods, and sometimes environmental friendly procedures and fewer waste products. The change in the production of nucleosidic compounds with respect to the non-nucleosidic ones leads to a reduction of the synthetic work, decreases the costs of the production of the new molecules, and, decreasing the amount of waste, reduces the impact on pollution and on the costs of side-product disposal.

### 2.3. Molecular Modelling

Currently, several X-Ray or cryo-EM structures of ARs in complex with nucleoside agonists are available, in particular of the A_2A_ [102,103,104] and A_1_ [105] AR subtypes. In contrast, structural experimental data depicting the interaction between non-nucleoside agonists and the ARs are still lacking. Studies were reported describing the effect of mutations of the binding site (or its proximity) residues on the affinity and/or efficacy of AR ligands, in some cases with the comparison of the effects on nucleoside- and non-nucleoside agonist activity. These works suggested different ligand–receptor recognition patterns for these two families of AR agonists. Molecular modelling studies were hence performed to interpret these data and to simulate the potential non-nucleoside agonist conformations within the AR binding cavity [36,37,63,64,68,106,107,108,109]. 

**Figure 7:**
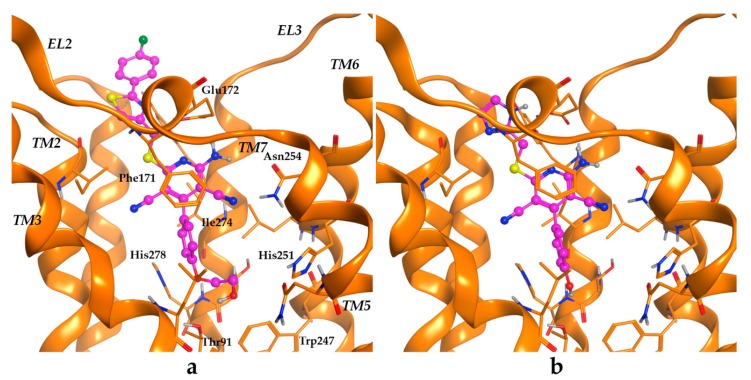
Putative binding mode of non-nucleoside agonists at the ARs. Panels (**a**,**b**) describe the potential binding mode of Capadenoson (**1**) and LUF5834 (**13**), respectively, at the A_1_AR. Key receptor residues involved in ligand–target interaction are indicated.

The docking conformation suggested by the majority of modelling studies presents the purine/pyrimidine scaffold of the agonists positioned analogously to the purine core of the nucleoside agonists (X-Ray/cryo-EM data), between a conserved phenylalanine in the extracellular loop (EL) 2 (i.e., Phe171 in the A_1_AR) and a conserved isoleucine in the transmembrane (TM) chain 7 (position 7.39 according to the Ballesteros and Weinstein numbering system [110]; in the A_1_AR, this position is occupied by Ile274 [111]). Figure 7 shows the putative binding mode of Capadenoson (**1**) and LUF5834 (**13**) at the A_1_AR. The exocyclic amine has a polar interaction with a glutamate residue in EL2 (i.e., Glu172 in A_1_AR) that is conserved among all the AR subtypes apart from the A_3_AR. The same amine and the cyano group next to this function have a polar interaction with the AR conserved asparagine in the 6.55 position (i.e., Asn254 in the A_1_AR), while the aromatic ring directly linked to the heterocyclic core points toward the depth of the cavity. The thioarylalkyl chain is oriented toward the extracellular environment. This arrangement appears the most suitable to interpret the activity of Capadenoson analogues at the A_1_AR or the Otsuka Pharmaceuticals pyrimidine-based A_2A_AR agonists, since the aromatic ring directly linked to the heterocyclic core generally presents small substituents that may find space within the depth of the binding cavities with no detrimental effect on the compound arrangement. 

The bulky thioarylalkyl chains of these compounds are located at the entrance of the binding cavities, where large amounts of space are available to accommodate these groups. The role of these chains to provide selectivity for a specific AR subtype seems critical since the kind of heterocycle inserted within this substituent modulates AR affinity. In fact, while the presence of an imidazole leads to compounds with nanomolar affinity for A_1_, A_2A_, and A_2B_ subtypes, its replacement with a thiazole generally enhances A_1_AR selectivity. The presence of a pyridyl group appears favorable to improve A_2A_AR affinity. Modeling studies have not clarified this feature since the thioarylalkyl chain may adopt several arrangements at the entrance of the binding cavity, with a consequent difficulty in the interpretation of the affinity data. The effect of the second cyano group (in the para-position with respect to the exocyclic amine) of the pyridines on the binding affinity has not been totally clarified as well, since compounds lacking this group (i.e., the corresponding pyrimidine derivatives) are endowed with nanomolar affinity for the respective ARs. Compounds bearing this function were suggested to have an additional interaction with a conserved histidine residue in position 7.43 (i.e., His278 in the A_1_AR).

According to the above-described arrangement, the exocyclic amine would occupy the analogue position of the 6-amine group of nucleoside agonists. Hence, modifications of this group with the insertion of further substituents could follow the “rules” depicted by Structure-Activity Relationship (SAR) analyses of nucleoside analogues. As an example, the insertion of alkyl/cycloalkyl groups in the 6-amine of adenosine led to the development of the selective A_1_AR agonist cyclopentyladenosine (CPA). Analogously, the modification of the A_1_AR agonist Capadenoson (**1**) through the replacement of the exocyclic amine with a pyrrolidinyl group led to the development of Neladenoson (**2**), another A_1_AR agonist with improved selectivity versus the A_2B_AR (see EC_50_ data of **1** and **2**, Table 1). However, this rule appears to not always be respected in the case of non-nucleoside derivatives, since some above-described antagonists presenting analogue docking conformations were modified with the insertion of alkyl groups in the exocyclic amine but the obtained affinities at ARs were generally lower respect to the unmodified compounds [62]. 

**Figure 8:**
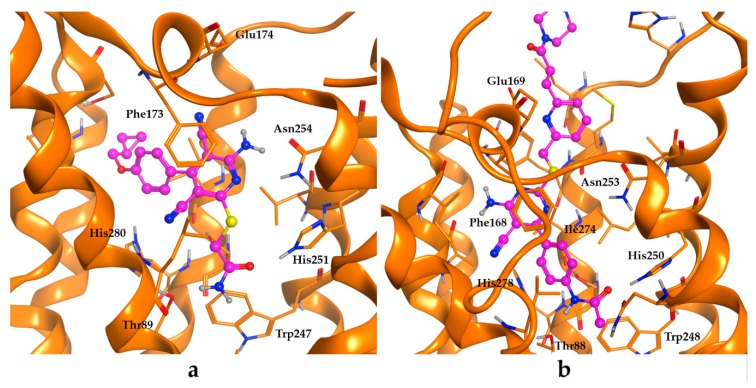
Putative binding mode of non-nucleoside agonists at the ARs. Panels (**a**,**b**) describe the potential alternative binding mode of non-nucleoside agonists at the A_2B_AR (BAY 60–6583, **25**) and at the A_2A_AR (**33**), respectively. Key receptor residues involved in ligand–target interaction are indicated.

Further arrangements were reported, with some relevant differences with respect to the above-described one. At the A_2B_AR, docking studies of pyridine-based agonists suggested an additional arrangement with the compounds (Figure 8a) [64,68,106,108].

This docking conformation makes the heterocyclic core still be located in the center of the binding cavity, but the phenyl ring directly linked to it points toward the extracellular environment, while the thioarylalkyl chain is inserted in the depth of the cavity. The exocyclic amine again has a polar interaction with a glutamate residue in EL2 (i.e., Glu174 in A_2B_AR), while the polar interaction with the conserved asparagine 6.55 (Asn254 in the A_2B_AR) is given by the exocyclic amine and the nitrogen atom of the pyridine core, while the cyan group next to the exocyclic amine points toward the extracellular space. However, this arrangement appears possible only for derivatives presenting a small thioarylalkyl group. A further docking conformation was observed at the A_2A_AR for the Otsuka Pharmaceutical pyrimidine-based agonists (Figure 8b) [109]. This conformation is an upside-down version of the general binding mode described above, with the thioarylalkyl group externally located and the phenyl ring in the depth of the cavity. The exocyclic amine interacts with TM2 residues, while the cyano group points toward the conserved histidine in the 7.43 position (i.e., His278 in the A_2A_AR). The polar interaction with the conserved asparagine in the 6.55 position (i.e., Asn253 in the A_2A_AR) is given by one of the nitrogen atoms of the heterocyclic core. This arrangement could be in agreement with the affinity data, but its occurrence apparently depends on the arrangement of the EL2 glutamate residue (i.e., Glu169 in A_2A_AR) [109].

Experimental (X-Ray/cryo-EM) data would be of great use for interpreting the biological activity of these compounds and for the design of further simplified agonists with high affinity, i.e., for the A_3_AR, the only AR subtype at which the above-described non-nucleoside agonist presents low or null activity.

## 3. Conclusions

Agonists of the ARs were only analogues of nucleoside adenosine until the discovery of the agonist properties of some pyridine derivatives in the early 2000s. This discovery prompted researchers to identify new non-nucleoside molecules endowed with increased activity and/or selectivity compared to the various AR subtypes, due to their easy synthesis with respect to the production of nucleosides, especially from the industrial point of view. 

This field appears promising, since on the one hand, selective A1, A2A, and A2B AR non-nucleoside agonists have been found only with two heterocyclic scaffolds (pyridine and pyrimidine), On the other hand, selective non-nucleoside agonists of the A_3_AR subtype have not been yet identified, leaving this goal still open. Finally, the understanding of the binding mode of non-nucleoside structures at the AR cavities could significantly help the design of novel agonists based on a simplified structure, with advantages due to the possibility of exploring different scaffolds and various substituents, which may also lead to good drug-like properties. 

## Figures and Tables

**Table 1:** Binding affinity (K_i_) and potency (EC_50_) data of selected pyridine-based non-nucleoside agonists of the ARs.

Compound	Affinity Data (K_i_ nM, or % Radioligand Displ.)				Potency Data (EC_50_ nM)		
	A_1_AR	A_2A_AR	A_2B_AR	A_3_AR	A_1_AR	A_2A_AR	A_2B_AR
**1** [36,41]	1.4 ^a,d^	0% ^b,e^	2.5% ^a,f^	1.2% ^a,g^	0.66 ^c^	1400 ^c^	1.1 ^c^
**2** [50]	-	-	-	-	0.1 ^a^	670 ^a^	80 ^a^
**4** [36]	5.0 ^a,d^	5.8% ^b,e^	20% ^a,f^	19% ^a,g^	2.9 ^a^	-	-
**5** [36]	1.5 ^a,d^	-	-	-	-	-	-
**6** [36]	8.4 ^a,d^	0.0% ^b,e^	63% ^a,f^	27% ^a,g^	1.9 ^a^	-	-
**7** [36]	3.9 ^a,d^	-	-	-	-	-	-
**8** [36]	12 ^a,d^	-	-	-	-	-	-
**9** [31]	2.4 ^a,d^	28 ^b,e^	-	171 ^b,h^	-	-	19 ^a^
**10** [31]	2.0 ^a,d^	105 ^b,e^	-	74 ^b,h^	-	-	34 ^a^
**11** [31]	7.0 ^a,d^	214 ^b,e^	-	24 ^b,h^	-	-	9 ^a^
**12** [31]	4.4 ^a,d^	21 ^b,e^	-	104 ^b,h^	-	-	10 ^a^
**13** [31,37]	2.6 ^a,d^	28 ^b,e^	-	538 ^b,h^	3.29 ^a^	16.2 ^b^	12 ^a^
**14** [60]	0.49 ^b,d^	71 ^b,e^	75 ^b,i^	42% ^b,h^	1.0 ^b^	-	-
**15** [32,39]	15 ^a,d^	23% ^a,e^	-	26% ^b,h^	0.7 ^a^	> 3000 ^a^	670 ^a^
**16** [32]	12 ^a,d^	25% ^a,e^	-	16% ^b,h^	-	-	-
**17** [32,39]	23 ^a,d^	37% ^a,e^	-	0% ^b,h^	0.5 ^a^	> 3000 ^a^	248 ^a^
**18** [32]	4.3 ^a,d^	21% ^a,e^	-	18% ^b,h^	-	-	-
**19** [32,39]	41 ^a,d^	8% ^a,e^	-	21% ^b,h^	2.7 ^a^	> 3000 ^a^	> 3000 ^a^
**20** [62]	57 ^a,d^	27% ^a,e^	-	29% ^a,h^	-	-	> 1000 ^a^
**21** [62]	1.02 ^a,d^	93 ^a,e^	-	668 ^a,h^	-	-	> 1000 ^a^
**22** [62]	0.98 ^a,d^	31 ^a,e^	-	25% ^a,h^	-	-	> 1000 ^a^
**23** [62]	1.42 ^a,d^	24 ^a,e^	-	948 ^a,h^	-	-	> 1000 ^a^
**24** [62]	1.32 ^a,d^	67 ^a,e^	-	326 ^a,h^	-	-	> 1000 ^a^
**25** [39,63,64]	31% ^a,d^	2% ^a,e^	114 ^a,f^	8% ^a,h^	-	-	10 ^a^
**26** [64]	345 ^a,d^	1% ^a,e^	-	20% ^a,h^	-	-	38 ^a^
**27** [64]	83 ^a,d^	25% ^a,e^	-	1% ^a,h^	-	-	12.7 ^a^
**28** [64]	235 ^a,d^	764 ^a,e^	-	474 ^a,h^	-	-	9.5 ^a^
**29** [64]	338 ^a,d^	1% ^a,e^	-	1% ^a,h^	-	-	51 ^a^
**30** [64]	8.2 ^a,d^	221 ^a,e^	-	85 ^a,h^	-	-	11.7 ^a^

(^a^) human AR subtype stably expressed by CHO (Chinese hamster ovary) cells; (^b^) human AR subtype stably expressed by HEK (human embryonic kidney) 293 cells; (^c^) human AR subtype stably expressed by FlpIn-CHO cells; (^d^) displacement of [^3^H]DPCPX as radioligand; (^e^) displacement of [^3^H]ZM241385 as radioligand; (^f^) displacement of [^3^H]PSB603 as radioligand; (^g^) displacement of [^3^H]PSB11 as radioligand; (^h^) displacement of [^125^I]I-ABMECA as radioligand; (^i^) displacement of [^3^H]NECA as radioligand. For complete experimental details, please refer to the indicated references.

**Table 2:** Binding affinity (K_i_) and potency (EC_50_) data of selected pyrimidine-based non-nucleoside agonists of the ARs.

Cpd	Affinity Data (K_i_ nM, or % Radioligand Displacement)				Potency Data (EC_50_ nM)		
	A_1_AR	A_2A_AR	A_2B_AR	A_3_AR	A_1_AR	A_2A_AR	A_2B_AR
**31** [30,38]	-	-	-	-	-	2.8 ^b^	-
**32** [30,38]	-	-	-	-	-	2.5 ^b^	-
**33** [30,38]	-	-	-	-	-	3.0 ^b^	-
**34** [30,38]	-	-	-	-	-	3.3 ^b^	-
**35** [30,38]	-	-	-	-	-	2.9 ^b^	-
**36** [30,38]	-	-	-	-	-	1.8 ^b^	-
**37** [35]	4.8 ^a,c^	10% ^b,d^	3.5% ^a,e^	3.0% ^a,f^	12 ^a^	-	-
**38** [35]	14 ^a,c^	44% ^b,d^	2.5% ^a,e^	5.5% ^a,f^	3.9 ^a^	-	-
**39** [35]	2.4 ^a,c^	−2.0% ^b,d^	20% ^a,e^	24% ^a,f^	4.6 ^a^	-	-
**40** [35]	14 ^a,c^	4.5% ^b,d^	0% ^a,e^	4.5% ^a,f^	4.9 ^a^	-	-
**41** [35]	5.2 ^a,c^	2.9% ^b,d^	1.7% ^a,e^	−1.8% ^a,f^	4.6 ^a^	-	-
**42** [35]	1.8 ^a,c^	3.2 ^b,d^	3.5 ^a,e^	11 ^a,f^	1.9 ^a^	-	-
**43** [80]	1240 ^a,c^	>10,000 ^a,g^	-	>10,000 ^a,g^	490 ^a^	-	Inactive ^a^
**44** [80]	1945 ^a,c^	>10,000 ^a,g^	-	>10,000 ^a,g^	870 ^a^	-	Inactive ^a^
**45** [81]	555 ^a,h^	200 ^a,g^	-	978 ^a,i^	-	-	-

(^a^) human AR subtype stably expressed by CHO cells; (^b^) human AR subtype stably expressed by HEK 293 cells; (^c^) displacement of [^3^H]DPCPX as radioligand; (^d^) displacement of [^3^H]ZM241385 as radioligand; (^e^) displacement of [^3^H]PSB603 as radioligand; (^f^) displacement of [^3^H]PSB11 as radioligand; (^g^) displacement of [^3^H]NECA as radioligand; (^h^) displacement of [^3^H]CCPA as radioligand; (^i^) displacement of [^3^H]HEMADO as radioligand. For complete experimental details, please refer to the indicated references.
